# Synthesis of Nitrogen-Rich Polymers by Click Polymerization Reaction and Gas Sorption Property

**DOI:** 10.3390/molecules23071732

**Published:** 2018-07-16

**Authors:** Jing-Ru Song, Wen-Gui Duan, Dian-Peng Li

**Affiliations:** 1 School of Chemistry & Chemical Engineering, Guangxi University, Nanning 530004, Guangxi, China; 2 Guangxi Institute of Botany, Chinese Academy of Sciences, Guilin 541006, Guangxi, China; ldp@gxib.cn

**Keywords:** microporous organic polymers, nitrogen-rich, CO_2_ adsorption, H_2_ adsorption, I_2_ vapor sorption

## Abstract

Microporous organic polymers (MOPs) are promising materials for gas sorption because of their intrinsic and permanent porosity, designable framework, and low density. The introduction of nitrogen-rich building block in MOPs will greatly enhance the gas sorption capacity. Here, we report the synthesis of MOPs from the 2,4,6-tris(4-ethynylphenyl)-1,3,5-triazine unit and aromatic azides linkers by click polymerization reaction. Fourier transform infrared (FTIR) and solid-state ^13^C CP-MAS (Cross Polarization-Magic Angle Spinning) NMR confirm the formation of the polymers. CMOP-1 and CMOP-2 exhibit microporous networks with a BET (Brunauer–Emmett–Teller) surface area of 431 m^2^·g^−1^ and 406 m^2^·g^−1^ and a narrow pore size distribution under 1.2 nm. Gas sorption isotherms including CO_2_ and H_2_ were measured. CMOP-1 stores a superior CO_2_ level of 1.85 mmol·g^−1^ at 273 K/1.0 bar, and an H_2_ uptake of up to 2.94 mmol·g^−1^ at 77 K/1.0 bar, while CMOP-2, with its smaller surface area, shows a lower CO_2_ adsorption capacity of 1.64 mmol·g^−1^ and an H_2_ uptake of 2.48 mmol·g^−1^. In addition, I_2_ vapor adsorption was tested at 353 K. CMOP-1 shows a higher gravimetric load of 160 wt%. Despite the moderate surface area, the CMOPs display excellent sorption ability for CO_2_ and I_2_ due to the nitrogen-rich content in the polymers.

## 1. Introduction

During the last few decades, microporous organic polymers (MOPs) have been intensively investigated and applied in various fields such as heterogeneous catalysis [1], gas adsorption and separation [2,3], the adsorption of radioactive iodine [4], drug delivery [5,6] and photovoltaics [7,8,9] duo to the low density, large surface area, high thermal/chemical stability, functional surface, and diverse building blocks. Until now, numerous MOPs including crystalline covalent organic frameworks (COFs) [10], covalent triazine frameworks (CTFs) [11], benzimidazole-linked polymers (BILPs) [12], porous polymer networks (PPNs) [13], hyper-crosslinked polymers (HCPs) [14], conjugated microporous polymers (CMPs) [15], polymers of intrinsic microporosity (PIMs) [16], and porous aromatic frameworks (PAFs) [17] have been synthesized via different polymerization reactions and versatile synthons. Amorphous polymers have received more and more attention owing to their scale-up preparation, high stability, and controllable synthesis compared with crystalline networks.

The excessive emission of carbon dioxide is believed to be the main contribution to the global warming problem resulting from the burning of fossil fuels. Great effort has been devoted to exploring the technology for CO_2_ capture and storage. Recently, MOPs have generated enormous interest in the area of gas sorption for carbon dioxide, hydrogen, iodine, and methane because of their low weight and remarkable microporous framework [18,19,20]. To achieve higher gas uptake by MOPs, a large surface area, suitable pore size, and strong interaction are crucial and desired. The introduction of heteroatoms, especially nitrogen or oxygen, has been demonstrated to be feasible and effective to enhance adsorption load by means of stronger interaction between gas molecules and adsorbent [21,22,23]. Triazine-based building blocks that have inherent nitrogen atoms can not only combine metal ions for catalysis [24], but also provide polarizable sites for gas uptake. Pan et al. reported imide functionalized 1,3,5-triazine frameworks named TPIs@IC. The polymers had ultramicropores at 5.4–6.8 Å, which can adsorb 3.2 mmol·g^−1^ CO_2_ (273 K/1 bar) and 1.47 wt% H_2_ (77 K/1 bar) [25]. Bu et al. synthesized aminal-linked porous organic polymers (APOPs) with a moderate to high surface area (S_BET_ = 724–1402 m^2^·g^−1^), but significant gas adsorption (CO_2_: 4.54 mmol·g^−1^ at 273 K/1 bar; H_2_: 8.95 mmol·g^−1^ at 77 K/1 bar), especially high CO_2_ adsorption, and good selectivity over N_2_ at low pressure (0.15 bar) [26]. In 2015, Lotsch et al. reported amorphous CTFs synthesized at different temperatures. For these polymers, excellent CO_2_ (5.58 mmol·g^−1^) and H_2_ (2.12 wt %) adsorption capacities were observed [27]. In addition, many CIFs with microporous frameworks for gas storage and separation have been reported, such as the cCTF (S_BET_ = 1247 m^2^·g^−1^; CO_2_: 13.3 wt%), *fl*-CTFs (S_BET_ = 15–2862 m^2^·g^−1^; CO_2_: 1.27–4.28 mmol·g^−1^), CTF-TB-3 (S_BET_ = 612 m^2^·g^−1^; CO_2_: 16.84 wt%), and CTF-BIs (CO_2_: 21.68 wt%) [28,29,30,31]. Zhang et al. synthesized polymers with N-heterocyclic groups (NAPOPs). NAPOP-1 and NAPOP-3 exhibit high adsorption toward iodine (>240 wt%) and moderate CO_2_ adsorption [32]. Geng et al. constructed novel triazine-based TCMPs by Friedel–Crafts polymerization reaction [33]. Among them, TTPPA displays excellent reversible iodine vapor uptake of 4.90 g·g^−1^. Furthermore, the TCMPs show sensitive sensing ability toward *o*-nitrophenol. Zhang et al. synthesized a triptycene-based polymer (NTP) by modified Yamamoto-type Ullmann cross-coupling reaction. The NTP displays good gas uptake (CO_2_: 15.2 wt%; H_2_: 1.58 wt%) and high iodine sorption capacity (180 wt%) [34]. Liao et al. constructed NCMPs with high N content and ultramicroporosity, which showed 215 wt% iodine vapor uptake at ambient pressure [35]. Zhao et al. reported a new nitrogen-rich COF with two kinds of micropores that exhibited extremely high iodine uptake (481 wt%) [36]. 

**Scheme 1:**
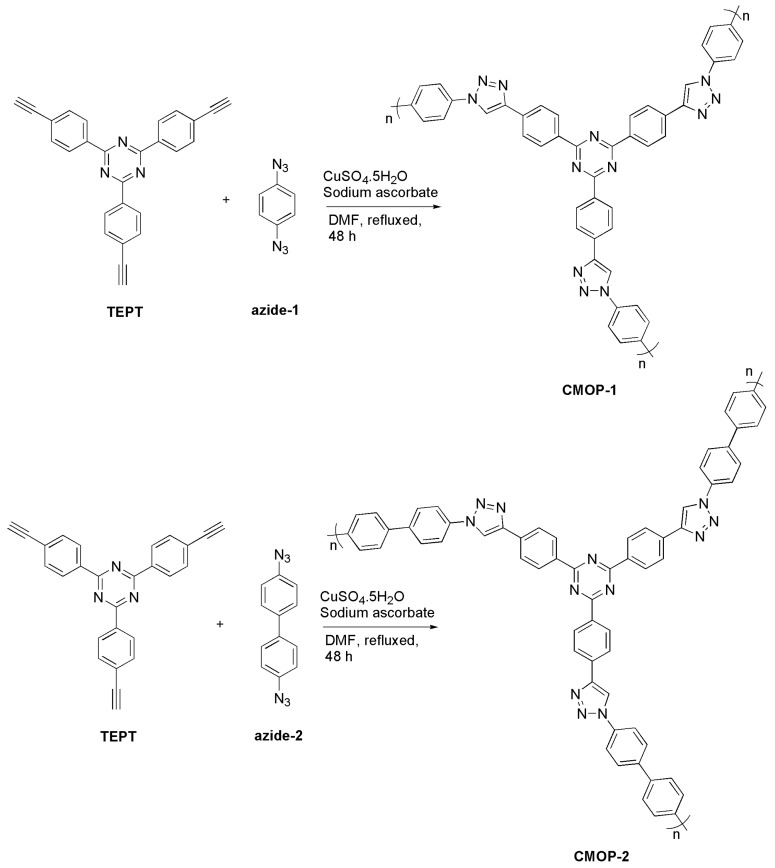
The synthetic route to themicroporous organic polymers.

The N-heterocycle derived from the efficient click reaction can further increase the nitrogen content for abundant sorption sites. Keeping this consideration in mind, we synthesized nitrogen-rich polymers named CMOPs from 2,4,6-tris(4-ethynylphenyl)-1,3,5-triazine (TEPT) and azides by click polymerization reaction (Scheme 1). The polymers were characterized by FTIR, solid state ^13^C CP-MAS, NMR, and elemental analysis (EA). Thermogravimetric analysis (TGA) showed the high thermal stability. Powder X-ray diffraction (PXRD) and scanning electron microscopy (SEM) confirmed the amorphous but uniform phase. Nitrogen adsorption–desorption isotherms at 77 K were investigated to explore the surface area, porosity, and pore size. In addition, adsorption measurements of CO_2_, H_2_, and I_2_ were researched to evaluate the gas sorption capacity.

## 2. Results

### 2.1. Synthesis and Character

The CMOPs were synthesized by click reaction in DMF solvent from TEPT and aromatic azides, according to the procedure in Scheme 1. The resulting brown powders were insoluble in common organic solvent such as tetrahydrofuran, dichloromethane, methanol, chloroform, acetone, and *N*,*N*-dimethylformamide. 

In the Fourier transform infrared (FTIR) spectra of CMOPs (Figure S1), characteristic peaks for TEPT at 1505 cm^−1^ and 3252 cm^−1^ attenuated dramatically, while peaks at 2106 cm^−1^ and 2095 cm^−1^ for azide-1 and azide-2 disappeared, and new peaks at 1617, 2921 cm^−1^ for CMOP-1 and 1612, 2927 cm^−1^ for CMOP-2 could be observed, confirming the occurring of click reaction and the formation of a triazole ring. The solid state ^13^C CP-MAS NMR measurements gave similar spectra for the two CMOPs (Figure S2). The peak at ~169 ppm could be attributed to the carbon atom in triazine ring, while the peak at ~ 146 ppm was ascribed to phenyl carbon, which was directly linked with the triazine ring. The main peaks at ~141 ppm and ~134 ppm originated from the two carbon atoms of the triazole ring. The broad signals at about ~ 128–110 ppm corresponded to the phenyl rings.

Thermogravimetric analysis in the N_2_ atmosphere showed that the polymers could keep thermal stability under 240 °C (CMOP-1) and 210 °C (CMOP-2). The first 5% weight loss was mainly due to the volatilization of solvent adsorbed in the polymers (Figure S3). Powder X-ray diffraction (PXRD) analysis in the angle range of 5° to 35° suggested the amorphous nature of the CMOPs (Figure S4). We considered that the relative strong peaks may originate the light copper complex due to the interaction between Cu^2+^ and nitrogen atoms. Scanning electron microscopy (SEM) images revealed the uniform morphology (Figure S5).

### 2.2. Porosity

**Figure 1:**
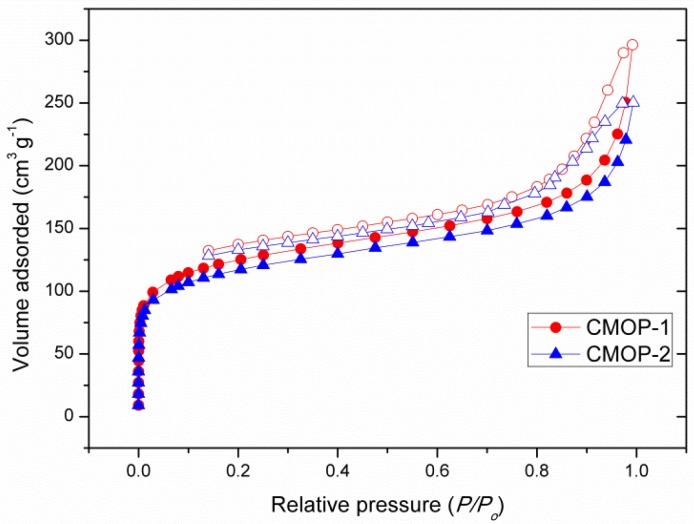
N_2_ adsorption–desorption isotherm at 77 K (filled: adsorption; empty: desorption).

The surface area and porosity of CMOPs was investigated by N_2_ adsorption–desorption measurements at 77 K. The results were illustrated in Figure 1. CMOP-1 and CMOP-2 exhibited type-I isotherms that showed a sharp adsorption at relative low pressure (*P*/*P_o_* < 0.05), indicating a permanent microporous network. The Brunauer–Emmett–Teller (BET) surface areas were calculated to be 431 m^2^·g^−1^ and 406 m^2^·g^−1^, while the microporous areas were 209 m^2^·g^−1^ and 188 m^2^·g^−1^. The total pore volumes of CMOP-1 and CMOP-2 were 0.458 cm^3^·g^−1^ and 0.387 cm^3^·g^−1^, with 0.09 cm^3^·g^−1^ and 0.08 cm^3^·g^−1^ microporous volume. The pore size distribution was estimated on the basis of the density functional theory (DFT) model. It revealed that the CMOPs exhibited two main distribution at 0.6 nm, 1.2 nm, and 0.7, 1.2 nm for CMOP-1 and CMOP-2, indicating the existence of ultramicroporosity (<1 nm), which was consistent with the microporous N_2_ sorption isotherm (Figure S6). Although the azide-2 molecule is longer than that of azide-1, the pore sizes were almost the same due to the disordered overlap in the formation of the porous network.

### 2.3. Gas Sorption Capacity

**Figure 2:**
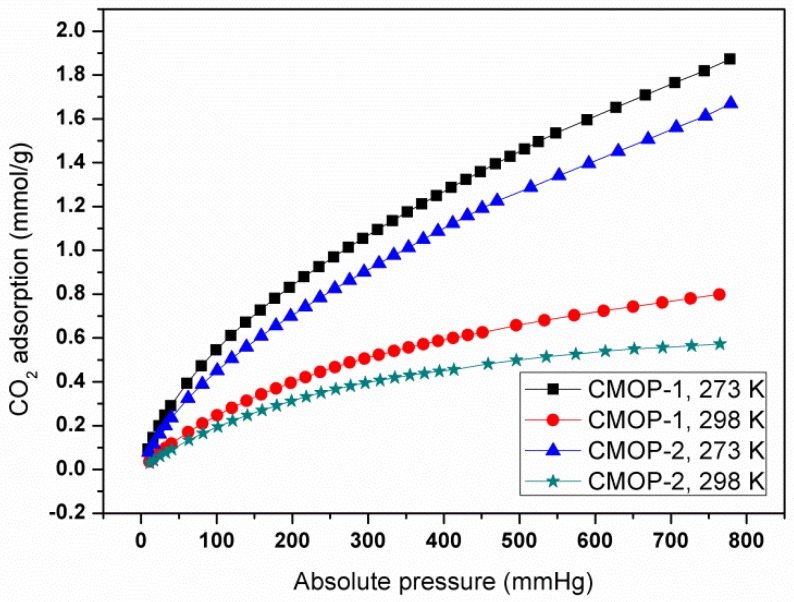
CO_2_ adsorption isotherm at 273/298 K.

The microporous framework with a narrow pore size distribution and nitrogen-rich character inspired us to explore CO_2_ adsorption capacity. As shown in Figure 2, CMOP-1 exhibited a higher CO_2_ adsorption ability of 1.85 mmol·g^−1^ (8.2 wt%) at 273 K and 0.80 mmol·g^−1^ (3.5 wt%) at 298 K, while CMOP-2, with its smaller surface area, showed a slightly lower uptake to 1.64 mmol·g^−1^ (7.3 wt%) and 0.57 mmol·g^−1^ (2.5 wt%) at 1.0 bar, which was superior to many heteroatom-rich polymers such as FPOP-1 (S_BET_: 538 m^2^·g^−1^, 6.3 wt%), NOP-55 (S_BET_: 526 m^2^·g^−1^, 8.6 wt%), CPOP-22 (S_BET_: 440 m^2^·g^−1^, 6.9 wt%), and CuPor-BPDC (S_BET_: 442 m^2^·g^−1^, 5.5 wt%) [37,38,39,40], but lower than other triazine-based polymers [26,27,28,29,30,31].

**Figure 3:**
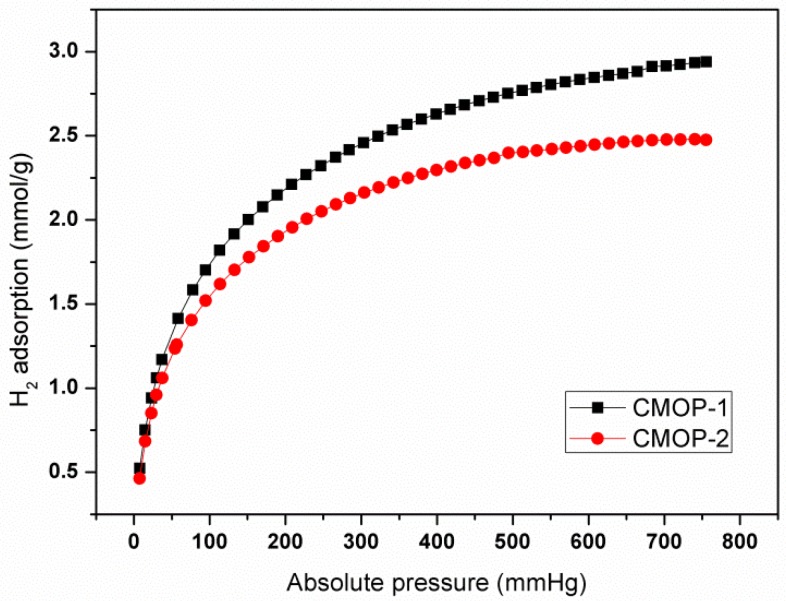
H_2_ adsorption isotherm at 77 K.

In addition, H_2_ uptake was also investigated at 77 K (Figure 3). The adsorption capacity was increasing along with the pressure, and then increased up to saturation. The maximum uptakes were 2.94 mmol·g^−1^ (0.6 wt%) and 2.48 mmol·g^−1^ (0.5 wt%) for CMOP-1 and CMOP-2. The moderate capacity originated from the low surface area. 

**Figure 4:**
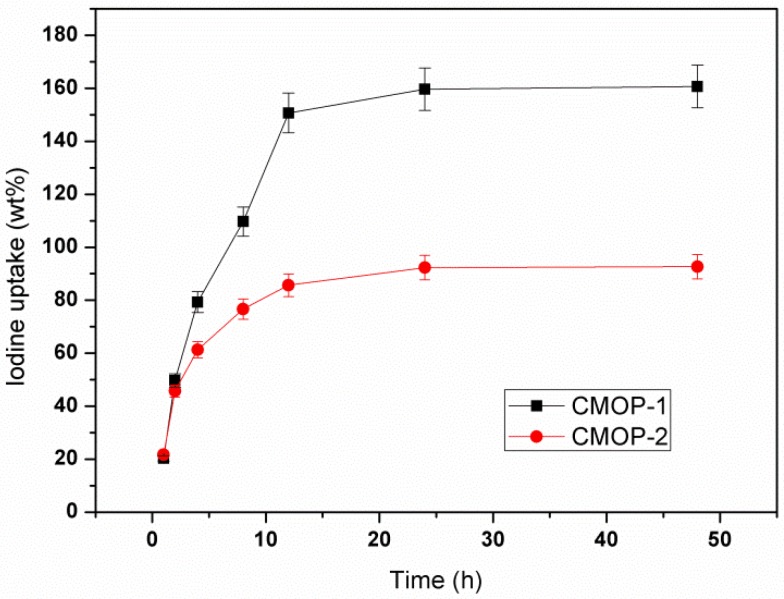
The uptake of I_2_ vapor at 353 K.

It was found that nitrogen-rich polymer favored the sorption toward iodine, owing to the stronger interaction between nitrogen atom and electron-deficient I_2_ [4,32,33]. The images in Figure S7 showed the color change of I_2_-cyclohexane (1 mg·mL^−1^) solution after adding 25 mg of CMOP-1 and CMOP-2. As time passed, the pink color of I_2_-cyclohexane solution faded clearly, indicating the good removal efficiency. To evaluate the I_2_ uptake ability of CMOPs, the sorption of I_2_ vapor was investigated at 353 K. The solid powder was placed in an open bottle that was exposed to vapor of I_2_ in a sealed container. As shown in Figure 4, the gravimetric uptake was gradually increasing, and then reached a plateau after about 24 h. The sorption ability was 160 wt% and 93 wt% for CMOP-1 and CMOP-2. The larger capacity of CMOP-1 was mainly attributed to the larger surface area and greater amount of nitrogen atoms in the surface.

## 3. Materials and Methods

### 3.1. General Information

TEPT and azides were synthesized according to the methods in the literature [41,42,43]. All of the other reagents were purchased from J&K (J&K, ShangHai, China) and used without further purification. 

Before all of the measurements, polymers were dried under vacuum at 120 °C overnight. FTIR spectra were recorded on a Thermo-Nicolet iS10 spectrometer using KBr discs (Thermo Scientific Co., Ltd., Madison, WI, USA). Solid state ^13^C CP-MAS NMR spectra data were collected on a BRUKER AVANCE III HD 400MHz NMR spectrometer (Bruker, Leipzig, Germany). Elemental analysis for C, H, and N were carried out with Elementar VarioMICRO Cube analyzer (Elementar, Frankfurt, Germany). Thermogravimetric analysis was performed on SDT Q600 V20.9 Build 20 (TA Instrument, New Castle, DE, USA) with a temperature ramping rate of 10 °C·min^−1^ from 30 °C to 850 °C. Scanning electron microscopy (SEM) was performed on ZEISS Evo 18 scanning electron microscope at 20.0 KV (Zeiss, Oberkochen, Germany). Powder X-ray diffraction (PXRD) data were recorded on Bruker D8 Advance (Bruker, Leipzig, Germany), from 5° up to 35° with 4°/min increment. Nitrogen adsorption–desorption isotherms were carried out with a Demo 2020 instrument at 77 K (Micromeritics Instrument Corp., Norcross, GA, USA). Carbon dioxide adsorption isotherms were measured at 273 K and 298 K on 3Flex Version 4.02 (Micromeritics Instrument Corp., Norcross, GA, USA). Hydrogen adsorption isotherms were conducted at 77 K on Quadrasorb SI (Quantachrome, FL, USA). 

### 3.2. Synthesis

CMOP-1: Under nitrogen atmosphere, TEPT (80 mg, 0.209 mmol), azide-1 (51 mg, 0.318 mmol), CuSO_4_·5H_2_O (15 mg, 0.06 mmol), sodium ascorbate (13 mg, 0.05 mmol), and dry DMF (7 mL) were placed in a flask, and then heated to reflux for 48 h. The resulting brown solid was filtrated and washed with water, methanol, and DCM. The residue was stirred in distilled water overnight, and then filtrated and dried in vacuum (101 mg, 77%). Anal. calculated for (C_12_H_7_N_4_)_n_: C 69.54, H 3.41, N 27.04; found (C_12_H_7_N_4_·0.05SO_4_^2−^)_n_: C 57.37, H 4.17, N 18.75, S 0.733

CMOP-2: Under nitrogen atmosphere, TEPT (68 mg, 0.178 mmol), azide-2 (64 mg, 0.271 mmol), CuSO_4_·5H_2_O (14 mg, 0.05 mmol), sodium ascorbate (11 mg, 0.045 mmol), and dry DMF (7 mL) were placed in a flask, and then heated to reflux for 48 h. The resulting brown solid was filtrated and washed with water, methanol, and DCM. The residue was stirred in distilled water overnight, and then filtrated and dried in vacuum (106 mg, 80%). Anal. calculated for (C_15_H_9_N_4_)_n_: C 73.44, H 3.70, N 22.84; found (C_15_H_9_N_4_·0.01SO_4_^2−^)_n_ : C 63.20, H 4.27, N 15.49, S 0.433.

### 3.3. The Gravimetric Sorption of I_2_ Vapor

10 mg of polymer was placed in an open bottle (10 mL) and the total weight was weighed (W_0_, mg). The open bottle was then putted into a sealed vessel (50 mL) that contained 200 mg of iodine at 353 K in the oil bath. The open bottle was taken out and weighed after 1 h, 4 h, 8 h, 12 h, 24 h, and 48 h (W_i_, mg), respectively. The gravimetric sorption (wt%) was calculated using the following formula:

wt% = (W_i_ − W_0_)/10 × 100%

## 4. Conclusions

In conclusion, two nitrogen-rich triazine-based microporous organic polymers were designed and synthesized by the condensation of a 2,4,6-tris(4-ethynylphenyl)-1,3,5-triazine building block and aromatic azides linkers for the first time. The resulting polymers possessed an ultramicroporosity network, good thermal stability, and high nitrogen content (CMOP-1: 18.17 wt%; CMOP-2: 15.49 wt%, from EA). Despite the moderate surface areas of 431 m^2^·g^−1^ and 406 m^2^·g^−1^ based on nitrogen adsorption, CMOPs exhibited relatively high CO_2_ sorption and moderate H_2_ sorption capacity that was comparable to or superior than many POPs with similar surface area. We ascribed the excellent CO_2_ sorption capacity to nitrogen-rich content, which improved the dipole–quadruple interaction between CO_2_ and polymers. Furthermore, the CMOPs displayed significant gravimetric uptake ability toward I_2_ vapor up to 160 wt%. The successfully constructed networks confirmed the advantage of nitrogen atoms for gas capture, and inspired our great interest to explore more functional MOPs.

## Supplementary Materials

Supplementary materials are available online. Figure S1. FTIR spectra of TEPT, azides and CMOPs. Figure S2. 13C CP-MAS NMR spectra of CMOP-1 and CMOP-2. Figure S3. Thermogravimetric analysis (TGA) curves of CMOP-1 and CMOP-2. Figure S4. Powder X-ray diffraction (PXRD) spectra of CMOP-1 and CMOP-2. Figure S5. SEM images (a) CMOP-1, (b) CMOP-2. Figure S6. Brunauer–Emmett–Teller (BET) plot and pore size distribution (a)/(c) CMOP-1, (b)/(d) CMOP-2. Figure S7. Color change of iodine–cyclohexane solution (a) CMOP-1, (b) CMOP-2.
