# Supplementary material for: Synthesis of Nitrogen-Rich Polymers by Click Polymerization Reaction and Gas Sorption Property
Source: Molecules. 2018 Jul 16;23(7):1732. doi: 10.3390/molecules23071732 (PMC6100294; doi:10.3390/molecules23071732)
Supplement: Supplementary file 1 [file molecules-23-01732-s001.pdf]

Supplementary Information

Article

Synthesis of Nitrogen-Rich Polymers by Click Polymerization Reaction and Gas Sorption Property

Jing-Ru Song [1,2,*], Wen-Gui Duan [1,*] and Dian-Peng Li [2]

[1] School of Chemistry & Chemical Engineering, Guangxi University, Nanning 530004, Guangxi, China
[2] Guangxi Institute of Botany, Chinese Academy of Sciences, Guilin 541006, Guangxi, China; ldp@gxib.cn
* Correspondence: songjingru@iccas.ac.cn (J.-R. S.); wgduan@gxu.edu.cn (W.-G. D.); Tel.: +86-771-209-7058 (W.-G. D.)

Received: 16 June 2018; Accepted: 13 July 2018; Published: date

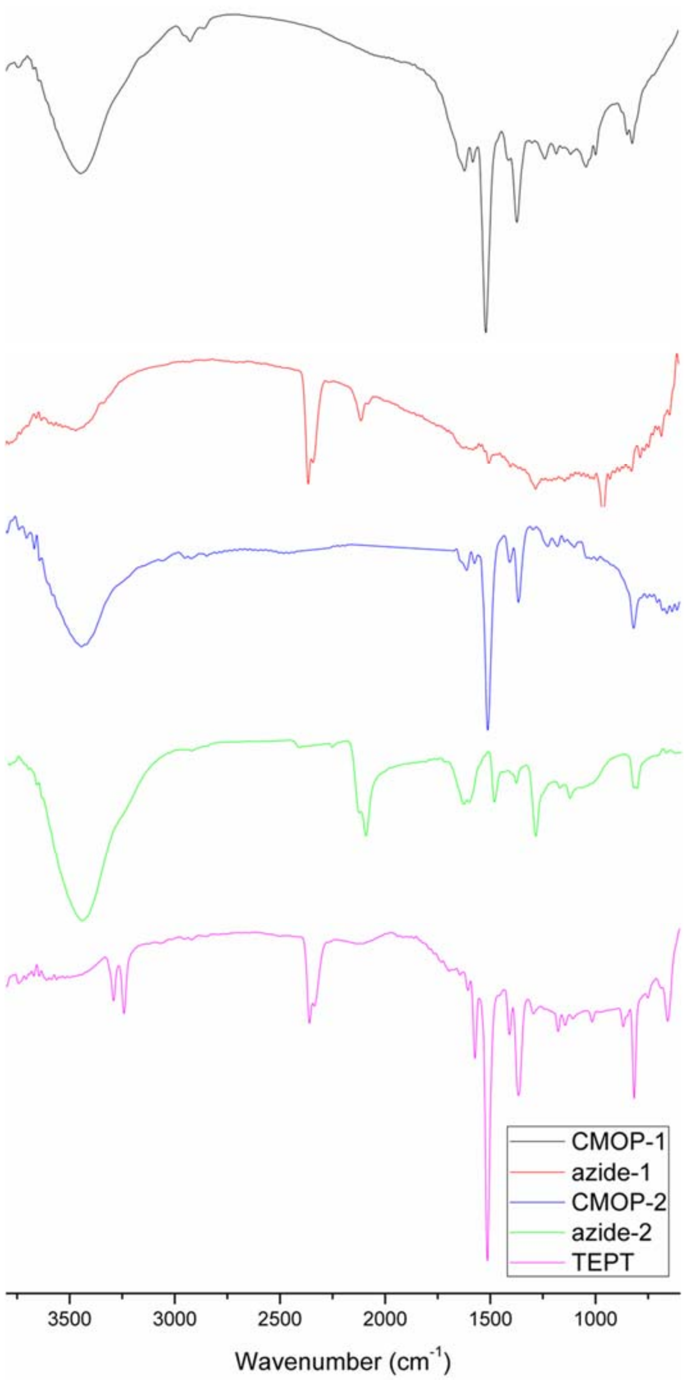

Figure S1. FTIR spectra of TEPT, azides and CMOPs.

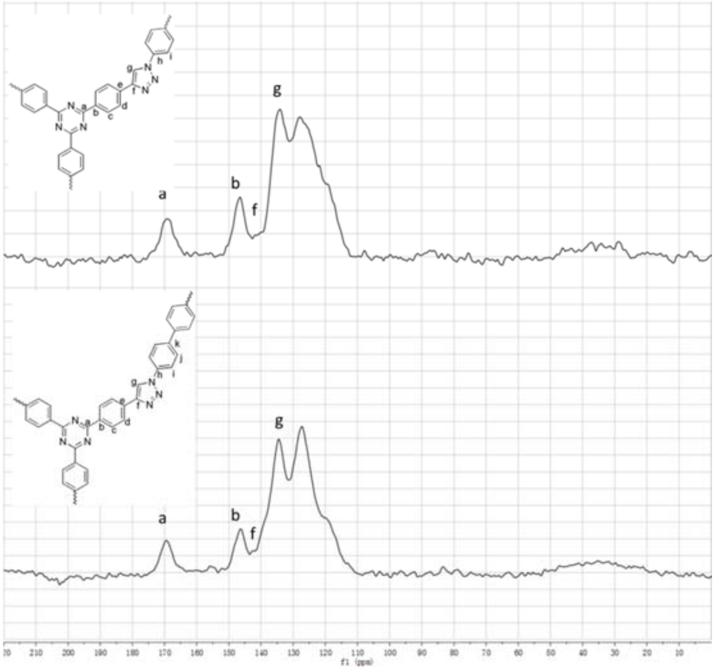

Figure S2. ^{13}C CP-MAS NMR spectra of CMOP-1 and CMOP-2.

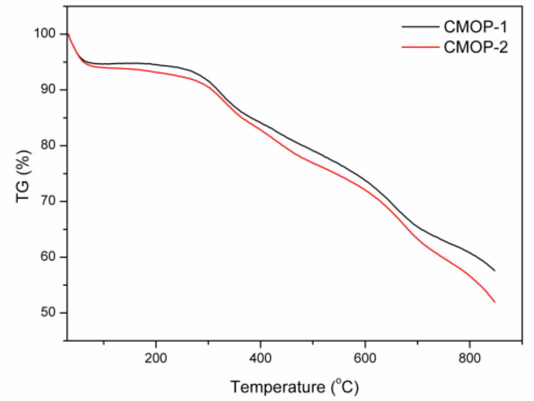

Figure S3. Thermogravimetric analysis (TGA) curves of CMOP-1 and CMOP-2.

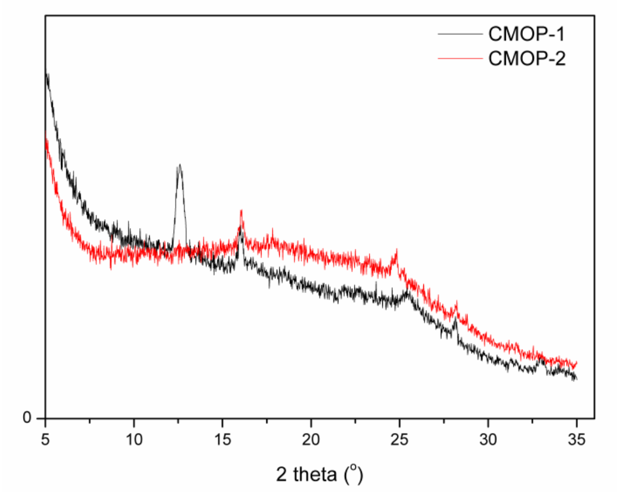

Figure S4. Powder X-ray diffraction (PXRD) spectra of CMOP-1 and CMOP-2.

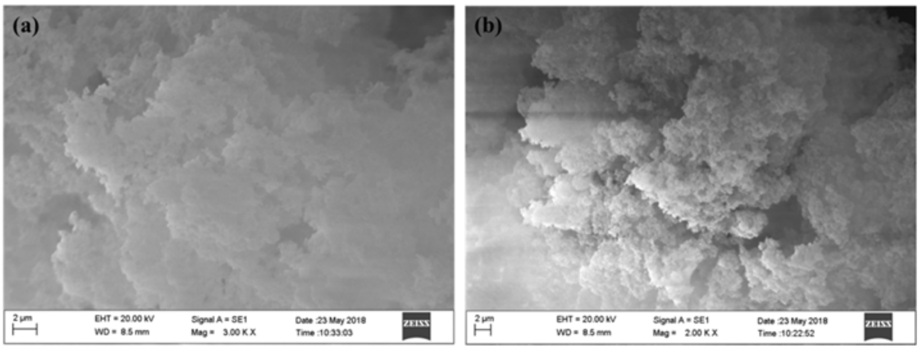

Figure S5. SEM images (**a**) CMOP-1, (**b**) CMOP-2.

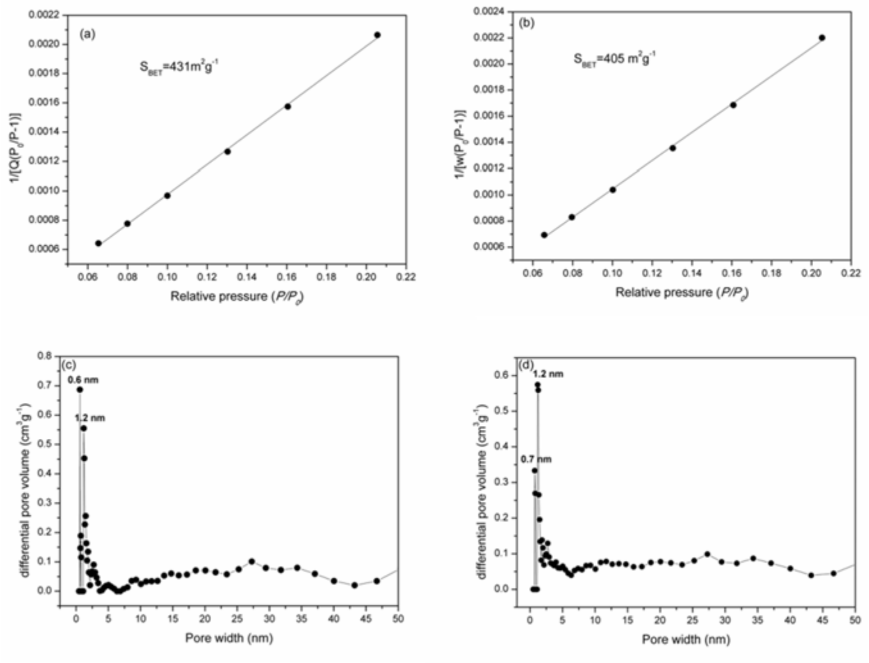

Figure S6. Brunauer–Emmett–Teller (BET) plot and pore size distribution (**a**)/(**c**) CMOP-1, (**b**)/(**d**) CMOP-2.

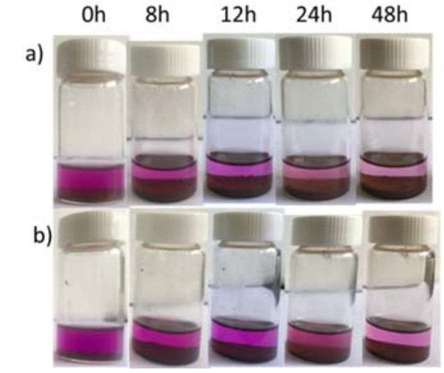

Figure S7. Color change of iodine–cyclohexane solution (**a**) CMOP-1, (**b**) CMOP-2.
